# Supplementary material for: Observation of topological hydrogen-bonding domains in physical hydrogel for excellent self-healing and elasticity
Source: Nat Commun. 2025 Mar 10;16:2371. doi: 10.1038/s41467-025-57692-y (PMC11894081; doi:10.1038/s41467-025-57692-y)
Supplement: Supplementary file 1 — Supplementary Information [file 41467_2025_57692_MOESM1_ESM.pdf]

Supplementary Information

Observation of Topological Hydrogen-bonding Domains in Physical Hydrogel for Excellent Self-Healing and Elasticity

Shaoning Zhang,[1,2,3] Dayong Ren,[3] Qiaoyu Zhao,[4] Min Peng,[2] Xia Wang,[2] Zhitao Zhang,[1] Wei Liu,[2,*] and Fuqiang Huang[1,2,3*]

[1] State Key Lab of Metal Matrix Composites, School of Materials Science and Engineering and Zhangjiang Institute for Advanced Study, Shanghai Jiao Tong University, 200240 Shanghai, China.

[2] School of Physical Science and Technology, ShanghaiTech University, Shanghai 200031, China.

[3] State Key Laboratory of High Performance Ceramics and Superfine Microstructure, Shanghai Institute of Ceramics, Chinese Academy of Sciences, Shanghai 200050, China.

[4] State Key Laboratory of Molecular Biology, Shanghai Institute of Biochemistry and Cell Biology, Center for Excellence in Molecular Cell Science, University of Chinese Academy of Sciences, Chinese Academy of Sciences, Shanghai 200031, China

* Corresponding authors:

Wei Liu, email: liuwei1@shanghaitech.edu.cn

Fuqiang Huang, email: huangfq@sjtu.edu.cn

Supplementary Information

The PDF file includes:

Figures S1 to S23

Tables S1 to S6

References S1 to S21

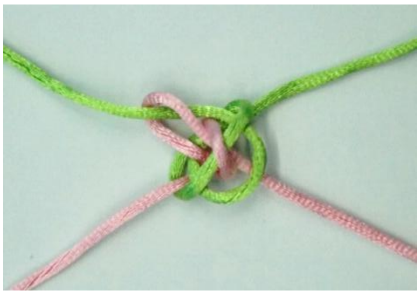

Supplementary Fig. 1 The photo of button knot. The presented button knot is made of two strings.

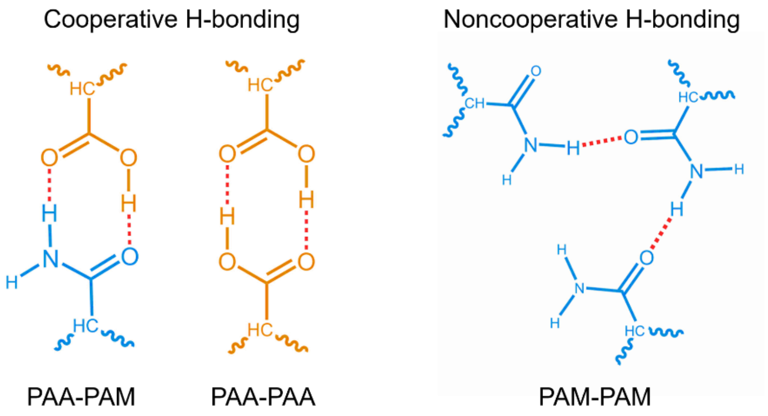

Supplementary Fig. 2 Schematic of H-bonding network consists of the cooperative H-bonding between carboxylic groups and amide groups in hydrogel and the noncooperative H-bonding between amide groups.

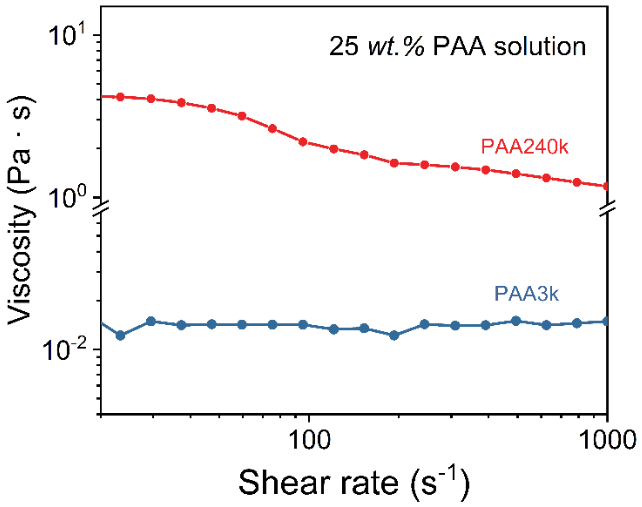

Supplementary Fig. 3 The complex viscosity of 25 *wt.%* PAA240k and PAA3k solution at various shear rates.

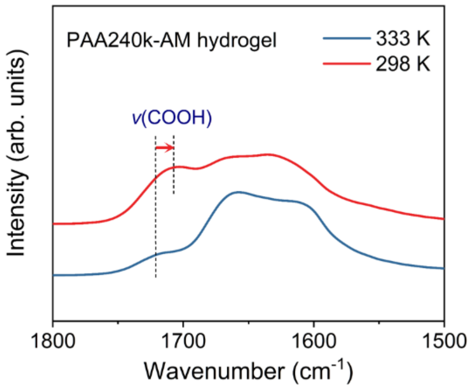

Supplementary Fig. 4 ATR-FTIR spectra of PAA240k-AM hydrogel at 333 K and 298 K.

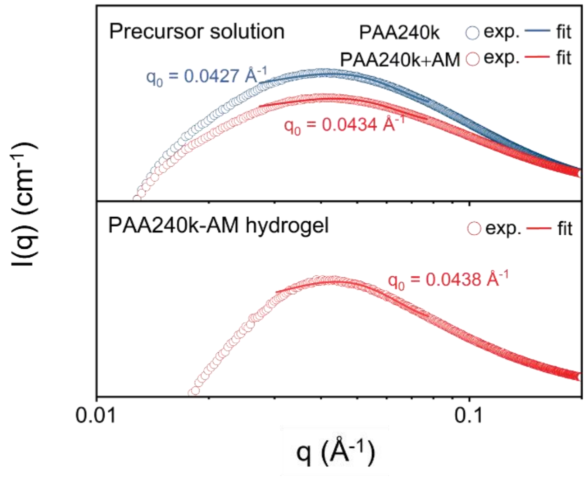

Supplementary Fig. 5 Small-angle x-ray scattering (SAXS) profiles of PAA240k solution, PAA240k-AM precursor solution and PAA240k-AM hydrogel with Gaussian fitting.

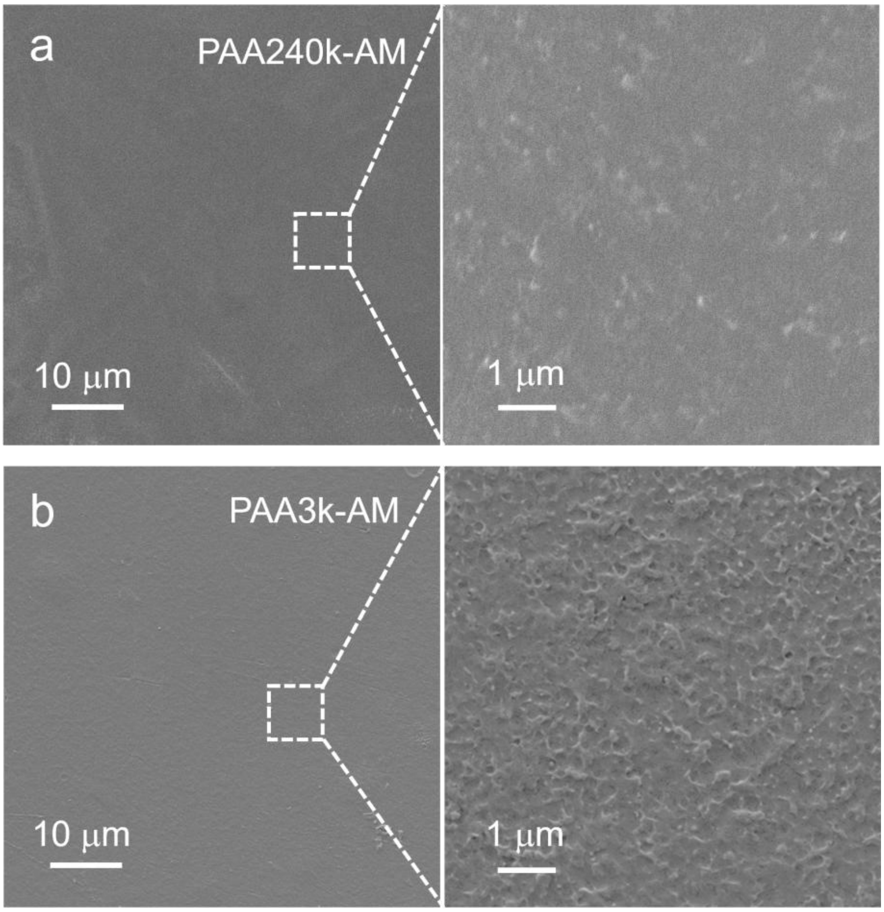

Supplementary Fig. 6 FESEM images of freeze-dried (A) PAA240k-AM and (B) PAA3k-AM hydrogels at different magnifications.

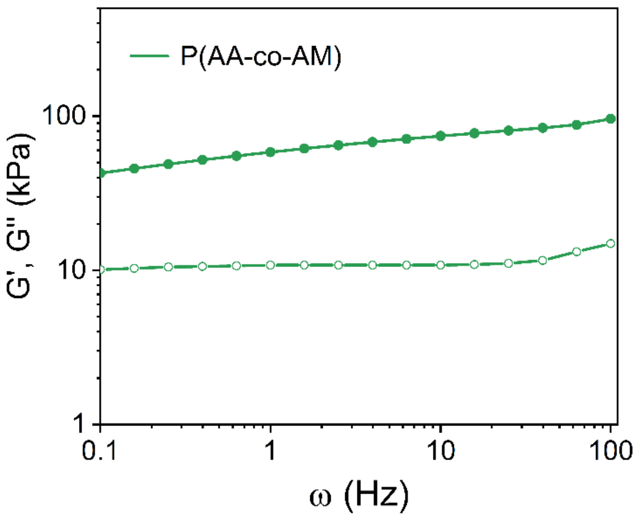

Supplementary Fig. 7 The storage modulus (G') and loss modulus (G'') of P(AA-co-AM) hydrogel.

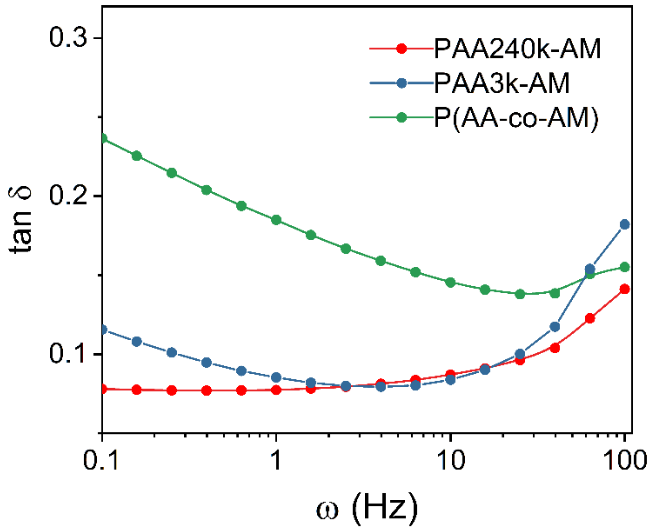

Supplementary Fig. 8 The loss factor (tan δ) of PAA240k-AM, PAA3k-AM and P(AA-co-AM).

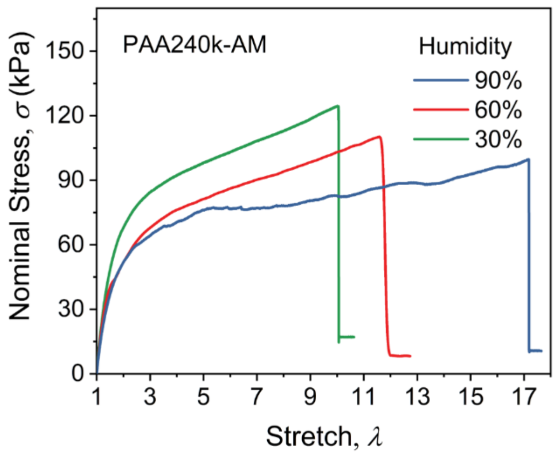

Supplementary Fig. 9 Stress-strain curves of PAA240k-AM at different humidities. The stretch rate is 2 min^{-1}.

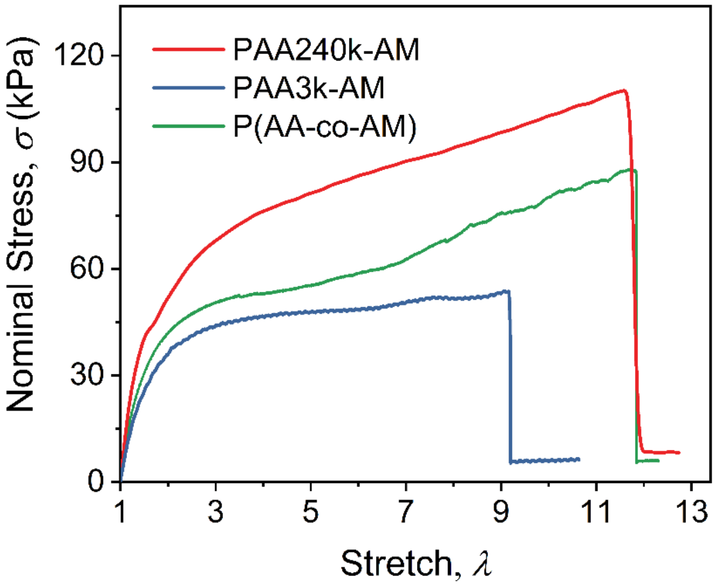

Supplementary Fig. 10 Stress-strain curves of PAA240k-AM, PAA3k-AM and P(AA-co-AM). The stretch rate is 2 min^{-1}.

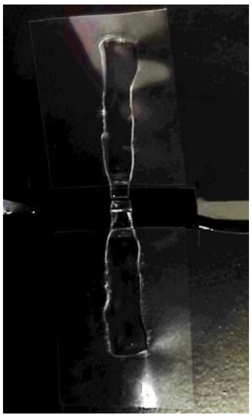

Supplementary Fig. 11 Photo of broken PAA240k-AM after the cutoff and self-healing for 5 hours.

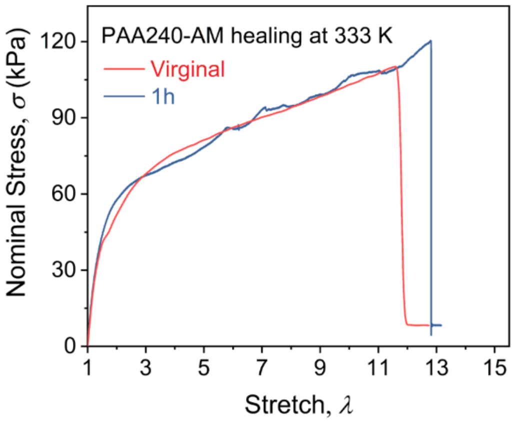

Supplementary Fig. 12 Stress-stretch curves of PAA240k-AM after different healing time at 333K. The stretch rate is 2 min^{-1}.

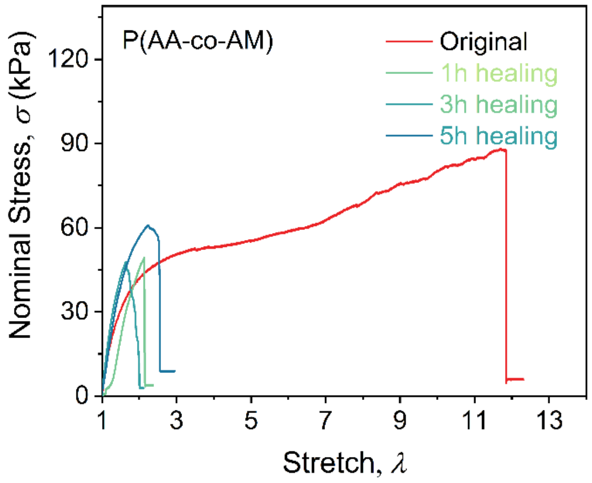

Supplementary Fig. 13 Stress-stretch curves of P(AA-co-AM) after different healing time. The stretch rate is 2 min^{-1}.

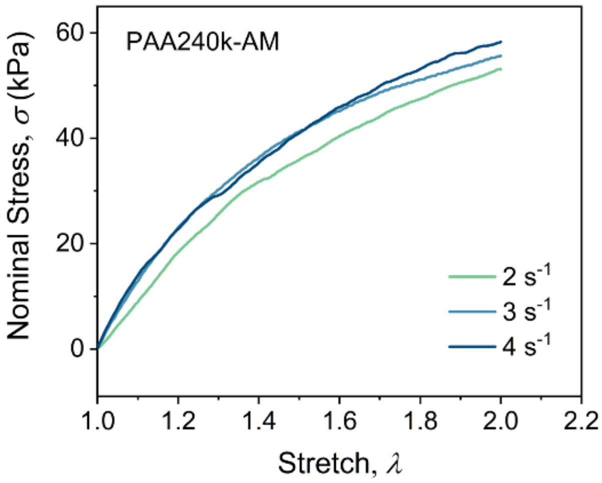

Supplementary Fig. 14 Stress-stretch curves of PAA240k-AM hydrogel at different stretching rates.

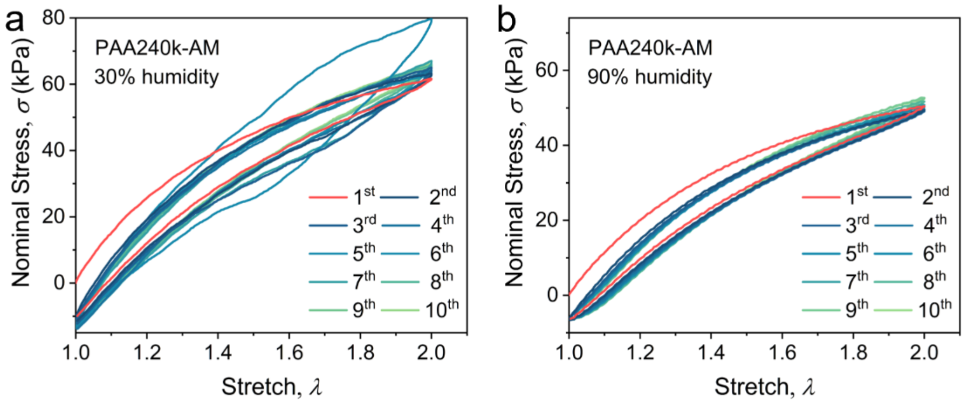

Supplementary Fig. 15 Successive stress-stretch curves of PAA240k-AM at (A) 30% humidity and (B) 90% humidity at λ = 2 for 10 cycles. The stretch rate is 3 min^{-1}.

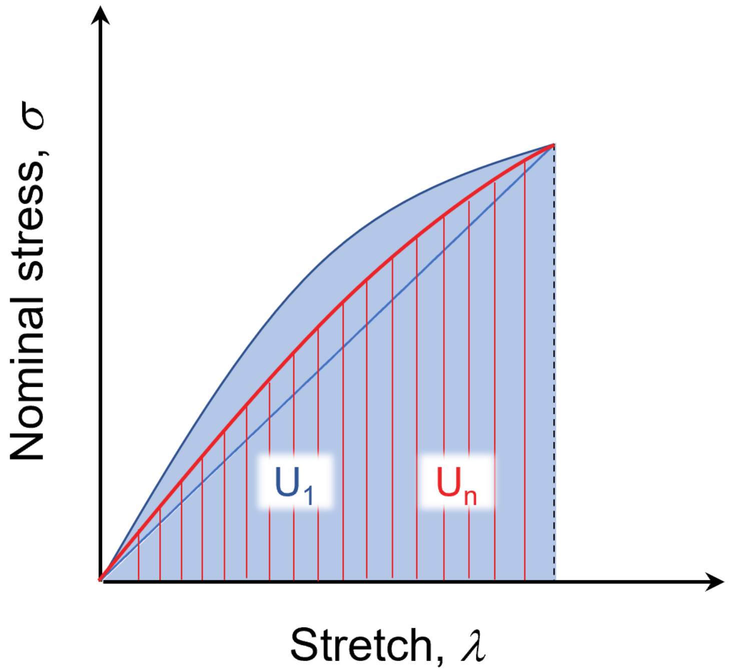

Supplementary Fig. 16 Quantification of instant reversibility. The reversibility is defined as the ratio of U_n to U_1, where U_i (i = 1, 2, 3, …, 10) is the area under the ith loading curve.

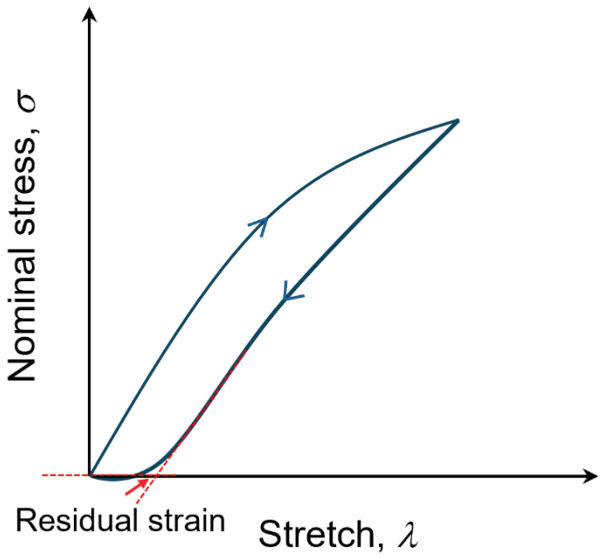

Supplementary Fig. 17 Quantification of residual strain. Residual strain is defined as the intersection of abrupt slope change of unloading curve.

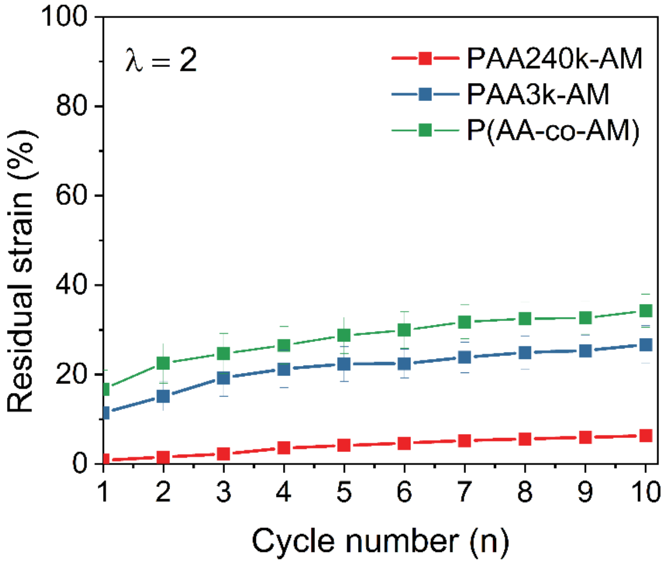

Supplementary Fig. 18 Residual strain of PAA-AM hydrogels cycling at stretch (*λ)* = 2 throughout 10 cycles.

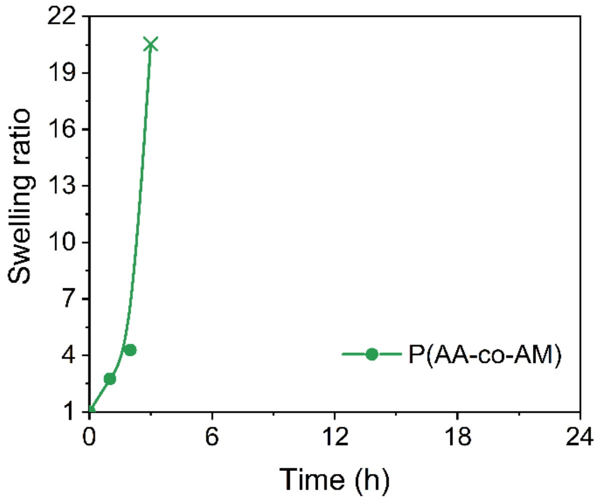

Supplementary Fig. 19 Weight change of P(AA-co-AM) hydrogel during swelling.

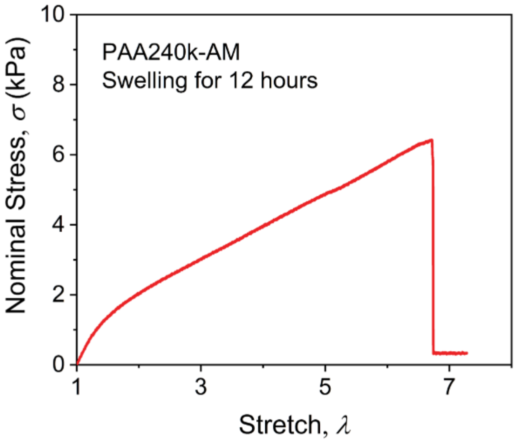

Supplementary Fig. 20 Stress-strain curve of swelled PAA240k-AM. The stretch rate is 2 min^{-1}.

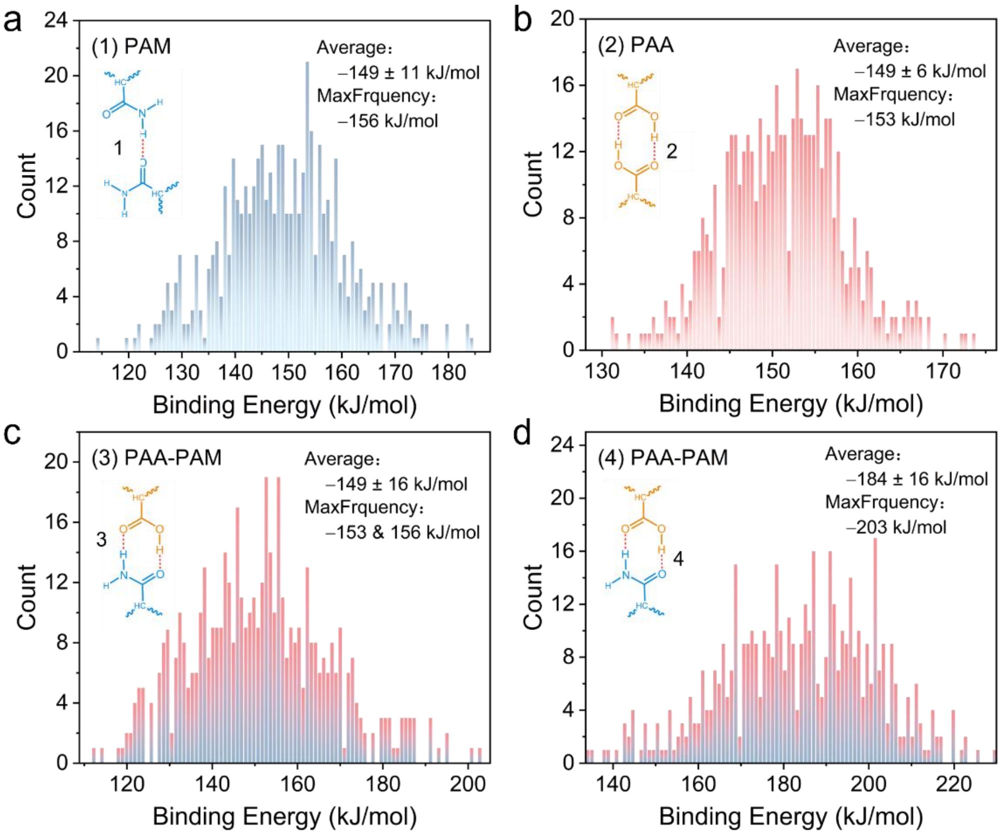

Supplementary Fig. 21 The distribution of bonding strength according to the molecular simulation.

(A) H-bonding between carbonyl and amino groups from amide groups in PAM.

(B) H-bonding between carbonyl and hydroxyl groups from carboxylic groups in PAA.

(C) H-bonding between carbonyl groups from PAA and amino groups from PAM.

(D) H-bonding between hydroxyl groups from PAA and carbonyl groups from PAM.

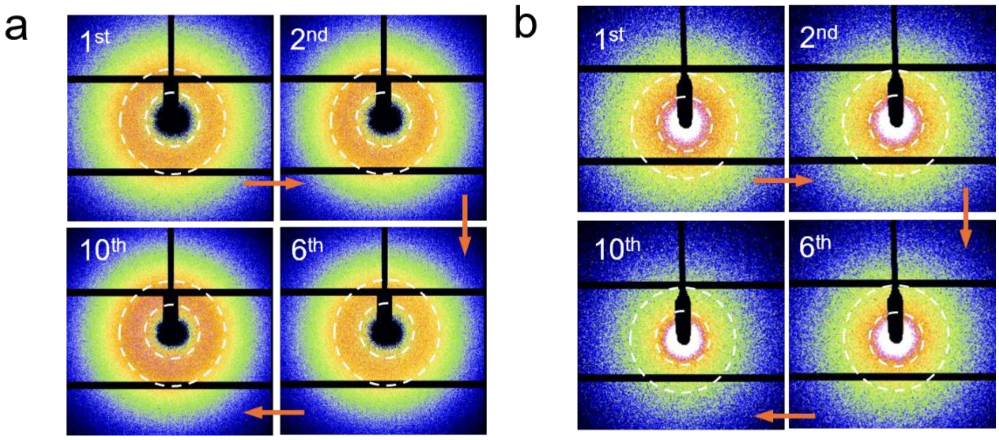

Supplementary Fig. 22 Time-resolved 2D SAXS patterns of (A) PAA240k-AM and (B) PAA3k-AM hydrogels during 10 loading cycles. The intensity in 2D SAXS patterns is normalized.

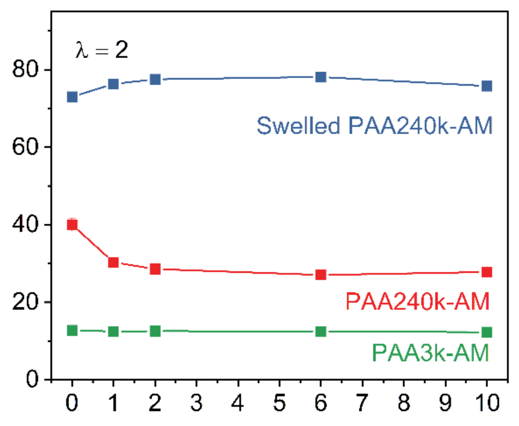

Supplementary Fig. 23 The correlation length fit from time-resolved SAXS profiles of PAA3k-AM, PAA240k-AM and swelled PAA240k-AM. The detailed fit results are listed in Table S3 and S4. The data range of fitting for PAA240k-AM is around 0.04 – 0.11 Å^{-1}. The data range of fitting for PAA3k-AM is around 0.01 – 0.12 Å^{-1}.

Supplementary Table 1 The Lorentzian peak fitting parameters of SAXS profiles of PAA240k solution, PAA240k+AM solution, PAA240k-AM hydrogel, PAA3k-AM and PAA3k-AM hydrogel.

Lorentzian peak fit	PAA240k solution	PAA240k +AM precursor	PAA240k -AM hydrogel	PAA3k solution	PAA3k+AM precursor
Scale	378.6±7.2	274.6±6.0	61.6±1.3	28.1±1.6	277.8±4.9
Background	601.6±7.3	515.2±6.2	80.6±1.4	66.3±1.6	29.6±1.2
Peak position (1/Å)	0.0414±0.0001	0.0420±0.0001	0.0435±0.0001	0.0642±0.0002	0.0182±0.0010
Peak HWHM (1/Å)	0.033±0.001	0.031±0.001	0.025±0.001	0.058±0.003	0.053±0.001
Fitting error	3.0	1.9	2.56	1.31	1.34

Supplementary Table 2 The Gaussian fitting parameters of SAXS profiles of PAA240k solution, PAA240k+AM solution and PAA240k-AM hydrogel.

Gaussian fitting	PAA240k solution	PAA240k +AM precursor	PAA240k -AM-hydrogel
Peak posit. (q_0)	0.0427±4E-5	0.0434±5E-5	0.0438±1E-4
Sigma	0.0201±1E-4	0.0189±1E-4	0.0177±2E-4
Scale	274.51±1.56	198.09±1.37	50.69±0.42
Background	701.69±1.68	588.92±1.48	90.47±0.46
Fitting error	15.97	12.17	12.83

Supplementary Table 3 The correlation length fitting parameters of SAXS profiles of PAA3k-AM hydrogel cycling at λ = 2.

**Correlation length fit**	**Virginal**	**1st cycle**	**2nd cycle**	**6th cycle**	**10th cycle**
Background	10.0±0.7	8.2±0.8	11.2±0.7	9.6±0.8	7.7±0.9
Lorentz scale	0.73±0.01	107.75±1.41	104.70±1.32	103.61±1.50	95.78±1.62
Porod scale	2.1E-7±4.6E-8	6.0E-5±9.5E-6	1.0E-4±1.5E-5	1.E-4±1.4E-5	8.3E-5±1.1E-5
Cor length	12.73±0.04	12.47±0.05	12.56±0.04	12.47±0.05	12.29±0.06
Porod expo	3.26±0.05	3.21±0.03	3.10±0.03	3.12±0.03	3.19±0.03
Lorentz expo	2.07±0.03	2.02±0.03	2.11±0.03	2.00±0.03	1.91±0.03
Fitting error	0.99	0.97	0.98	1.03	1.03

Supplementary Table 4 The Lorentzian peak fitting parameters of SAXS profiles of PAA240k-AM hydrogel cycling at λ = 2. The data of initial hydrogel is the same as that in table S1.

**Lorentzian peak fit**	**Virginal**	**1st cycle**	**2nd cycle**	**6th cycle**	**10th cycle**
Scale	61.6±1.3	138.4±2.6	167.5±2.9	198.7±3.4	168.3±3.1
Background	80.6±1.4	64.4±2.5	58.6±2.6	53.7±2.9	55.8±2.8
Peak position (1/Å)	0.0435±0.0001	0.0340±0.0003	0.0320±0.0003	0.0294±0.0004	0.0315±0.0003
Peak HWHM (1/Å)	0.025±0.001	0.033±0.001	0.035±0.001	0.037±0.001	0.036±0.001
Fitting error	2.56	1.29	1.53	1.07	1.13

Supplementary Table 5 The Lorentzian peak fitting parameters of SAXS profiles of swelled PAA240k-AM hydrogel cycling at $\lambda = 2$.

**Lorentzian peak fit**	**Virginal**	**1st cycle**	**2nd cycle**	**6th cycle**	**10th cycle**
Scale	380.0±0.5	454.1±0.5	561.9±0.5	1071.6±1.3	1115.8±3.0
Background	-0.7±0.2	-2.8±0.2	-5.9±0.2	-41.2±0.7	-60.1±1.5
Peak position (1/Å)	0.0165±0.0001	0.0169±0.0001	0.0167±0.0001	0.0166±0.0001	0.0168±0.0001
Peak HWHM (1/Å)	0.0137±0.0001	0.0131±0.0001	0.0129±0.0001	0.0128±0.0001	0.0132±0.0001
Fitting error	2.4	2.5	3.4	3.0	1.4

Supplementary Table 6 The performance comparisons of this work with related literatures.

Sample	λ_{max}	U_2/U_1 (%, λ_{cycle})	U_n/U_1 (%, cycle n)	Self-healing (%, condition)	Ref.
**Self-healing and elastic hydrogel**					
PAA240k-AM	11.5	87(2)	85(10)	100 (rt, 5h)	This work
**Non-self-healing hydrogel**					
SR-0.30	13.5	100(11)	100(100)	N/A	1
Highly entangled hydrogel	4.7	100(2.5)	N/A	N/A	2
PAM-CS-A	4.6	36(4)	30(10)	N/A	3
Hybrid	22	29(7)	29(7)	N/A	4
PVA-CBA/PAH	10.2	97(3)	91(5000)	100 (hot water, 24h)	5
D-hydrogel-0.15	8.5	48(2)	30(2)	N/A	6
CR-1	115.8	97(21)	97(100)	N/A	7
**Softening self-healing hydrogel**					
PVA/PAA-1/Fe-1.5	13.6	77(7)	52(10)	50 (rt, 12h)	8
PMAI-Zn-2	4	87(2)	64(10)	64 (rt, 4h)	9
PAM/SA-$Fe_{0.018}$	15	65(7)	65(5)	46 (343 K, 12h)	10
PHMA HA	24	35(8)	35(8)	30 (333 K, 12h)	11
G10H50 gelatin/pHEAA	9.2	64(5)	60(3)	35 (333 K, 12h)	12
PAA-GO-Ca^{2+}	26	19 (9)	19(2)	88 (rt, 20h)	13
PAA/betaine	16	49(11)	49(10)	100 (rt, 12h)	14
PAAFC-L	13	84(11, 60s)	82(20, 60s)	53 (rt, 48h)	15
PVA-$CBA_{0.12}$	6	68(3)	65(10)	100 (rt, 12h)	16
S-gel (AA/AM=0.2)	4.2	45(2)	45(2)	25 (1min)	17

Supplementary Table 6 Continued.

**Sample**	**λ_{max}**	**U_2/U_1 (%, λ_{cycle})**	**U_n/U_1 (%, cycle n)**	**Self-healing (%, condition)**	**Ref.**
PAA-Fe^{3+}/CS DN	13	75(3)	53(10)	50 (343 K, 24h)	18
$PMH_{1.2\%}$	18	65(11)	66(10)	62 (rt, 2h)	19
PAAm/PAA–1.1%Fe3+/NaCl	6.7	83(3)	63(10)	90 (rt, 24h)	20
PVA/CP DN	6.4	85(4)	N/A	14 (310 K, 12h)	21

References

1. Chang L*, et al.* Tough hydrogels with rapid self-reinforcement. *Science* **372**, 1078-1081 (2021).

2. Junsoo K, Guogao Z, Meixuanzi S, Zhigang S. Fracture, fatigue, and friction of polymers in which entanglements greatly outnumber cross-links. *Science* **374**, 212-216 (2021).

3. Yang Y, Wang X, Yang F, Shen H, Wu D. A Universal Soaking Strategy to Convert Composite Hydrogels into Extremely Tough and Rapidly Recoverable Double-Network Hydrogels. *Adv Mater* **28**, 7178-7184 (2016).

4. Sun J-Y*, et al.* Highly stretchable and tough hydrogels. *Nature* **489**, 133-136 (2012).

5. Li T, Li X, Yang J, Sun H, Sun J. Healable Ionic Conductors with Extremely Low-Hysteresis and High Mechanical Strength Enabled by Hydrophobic Domain-Locked Reversible Interactions. *Adv Mater* **35**, e2307990 (2023).

6. Lin P, Ma S, Wang X, Zhou F. Molecularly engineered dual-crosslinked hydrogel with ultrahigh mechanical strength, toughness, and good self-recovery. *Adv Mater* **27**, 2054-2059 (2015).

7. Li W*, et al.* Nanoconfined polymerization limits crack propagation in hysteresis-free gels. *Nat Mater* **23**, 131-138 (2023).

8. Liang Y, Sun X, Lv Q, Shen Y, Liang H. Fully physically cross-linked hydrogel as highly stretchable, tough, self-healing and sensitive strain sensors. *Polymer* **210**, 123039 (2020).

9. Dutta A, Ghosal K, Sarkar K, Pradhan D, Das RK. From ultrastiff to soft materials: Exploiting dynamic metal–ligand cross-links to access polymer hydrogels combining customized mechanical performance and tailorable functions by controlling hydrogel mechanics. *Chem Eng J* **419**, 129528 (2021).

10. Zheng Q, Zhao L, Wang J, Wang S, Liu Y, Liu X. High-strength and high-toughness sodium alginate/polyacrylamide double physically crosslinked network hydrogel with superior self-healing and self-recovery properties prepared by a one-pot method. *Colloids Surf, A* **589**, 124402 (2020).

11. Yang J*, et al.* Tough and Conductive Dual Physically Cross-Linked Hydrogels for Wearable Sensors. *Ind Eng Chem Res* **58**, 17001-17009 (2019).

12. Tang L*, et al.* Double-Network Physical Cross-Linking Strategy To Promote Bulk Mechanical and Surface Adhesive Properties of Hydrogels. *Macromolecules* **52**, 9512-9525 (2019).

13. Wang Y*, et al.* Tough but self-healing and 3D printable hydrogels for E-skin, E-noses and laser controlled actuators. *J Mater Chem A* **7**, 24814-24829 (2019).

14. Zhang W, Wu B, Sun S, Wu P. Skin-like mechanoresponsive self-healing ionic elastomer from supramolecular zwitterionic network. *Nat Commun* **12**, 4082 (2021).

15. Wu M*, et al.* Ultra elastic, stretchable, self-healing conductive hydrogels with tunable optical properties for highly sensitive soft electronic sensors. *J Mater Chem A* **8**, 24718-24733 (2020).

16. Fang X*, et al.* Dynamic Hydrophobic Domains Enable the Fabrication of Mechanically Robust and Highly Elastic Poly(vinyl alcohol)-Based Hydrogels with Excellent Self-Healing Ability. *ACS Mater Lett* **2**, 764-770 (2020).

17. Liang Y, Xue J, Du B, Nie J. Ultrastiff, Tough, and Healable Ionic-Hydrogen Bond Cross-Linked Hydrogels and Their Uses as Building Blocks To Construct Complex Hydrogel Structures. *ACS Appl Mater Interfaces* **11**, 5441-5454 (2019).

18. Wang X-H*, et al.* Strong and tough fully physically crosslinked double network hydrogels with tunable mechanics and high self-healing performance. *Chem Eng J* **349**, 588-594 (2018).

19. Liu L, Li X, Ren X, Wu Gf. Flexible strain sensors with rapid self-healing by multiple hydrogen bonds. *Polymer* **202**, 122657 (2020).

20. Li S, Pan H, Wang Y, Sun J. Polyelectrolyte complex-based self-healing, fatigue-resistant and anti-freezing hydrogels as highly sensitive ionic skins. *J Mater Chem A* **8**, 3667-3675 (2020).

21. Gong Z*, et al.* High-Strength, Tough, Fatigue Resistant, and Self-Healing Hydrogel Based on Dual Physically Cross-Linked Network. *ACS Appl Mater Interfaces* **8**, 24030-24037 (2016).
