# Supplementary material for: Observation of topological hydrogen-bonding domains in physical hydrogel for excellent self-healing and elasticity
Source: Nat Commun. 2025 Mar 10;16:2371. doi: 10.1038/s41467-025-57692-y (PMC11894081; doi:10.1038/s41467-025-57692-y)
Supplement: Supplementary file 2 — Description of Additional Supplementary Files [file 41467_2025_57692_MOESM2_ESM.pdf]

Description of Additional Supplementary Files

File name: Supplementary Movie 1
Description: Molecular dynamics of low-molecular-weight PAA aqueous solution (r.u. = 30) and high-molecular-weight-PAA aqueous solution (r.u. = 256).

File name: Supplementary Movie 2
Description: Stretching PAA3k-AM and PAA240k-AM healing for 5 hours by hand.

File name: Supplementary Movie 3
Description: PAA240kAM cycling at λ = 2 for 10 cycles.

File name: Supplementary Movie 4
Description: Broken PAA240k-AM after the cutoff and self-healing for 5 hours.

File name: Supplementary Movie 5
Description: Swelled PAA240k-AM cycling for 100 times.
